# Sensors Data Processing Using Machine Learning

**DOI:** 10.3390/s24051694

**Published:** 2024-03-06

**Authors:** Patrik Kamencay, Peter Hockicko, Robert Hudec

**Affiliations:** Faculty of Electrical Engineering and Information Technology, University of Zilina, 010 26 Zilina, Slovakia; peter.hockicko@feit.uniza.sk (P.H.); robert.hudec@feit.uniza.sk (R.H.)

Various sensors utilize computational models to estimate measured variables, and the generated data require processing. Data processing involves transforming data from a given format into a more usable and desirable form, rendering them more meaningful and informative. Machine learning (ML), deep learning (DL), and artificial intelligence (AI) have proven effective for this purpose. The entire process can be automated using machine learning algorithms, mathematical modeling, or various statistical techniques.

The aim of this Special Issue was to compile research on data processing through machine learning and deep learning. It features both original and review articles that address research and development in data processing using machine learning (ML) and deep learning (DL). These areas include solutions designed for smart devices.

The first paper [1] focuses on detecting toxicity in online discussions. The authors used classification models that incorporate machine learning methods to classify short texts on social networking sites into multiple degrees of toxicity. Their models used both classic methods of machine learning, such as naive Bayes and SVM (support vector machine), as well as ensemble methods, such as bagging and RF (random forest). The models were created using text data, which they extracted from social networks in the Slovak language. Finally, an application was created based on machine learning models, which can be used to detect the degree of toxicity of new social network comments, as well as for experimentation with various machine learning methods. The best results were achieved using an SVM—average value of accuracy = 0.89 and F1 = 0.79. This model also outperformed the ensemble learning by the RF and Bagging methods; however, the ensemble learning methods achieved better results than the naïve Bayes method.

The second paper [2] introduces a framework for evaluating segments of physical and digital infrastructure, which is designed to assess their features and readiness for facilitating the deployment of Connected and Automated Vehicles (CAVs). It delves into the equipment and methodology employed to collect and analyze the necessary data for automated scoring of infrastructure segments. The authors illustrate the assessment methodology using two types of data: connectivity and positioning data for evaluating the infrastructure’s connectivity and localization performance, and image data for road signage detection through a Convolutional Neural Network (CNN). The data collection and analysis were conducted in both urban and suburban settings. The primary communication challenge in the examined area is latency, particularly in infrastructure segments located at busy intersections or near various points of interest. The study observed lower localization accuracy in dense areas with large buildings and trees, limiting the visibility of localization satellites. To address the challenge of automated traffic sign recognition precision assessment, the authors proposed a CNN that achieved a precision rate of 99.7%.

The authors of [3] introduce an enhanced IoT-based system to assist teachers in managing classroom activities in adherence to COVID-19 restrictions. The system, which comprises three components—an entry Gate node, IoT nodes, and a server—comprehensively monitors the number of individuals in the classroom and their spatial distribution. The Gate node, positioned at the entrance, tracks individuals entering or exiting the room through door crossing detection, while IoT nodes, based on Arduino with NodeMCU modules and ultrasonic distance sensors, collect data on seat occupancy. The server, hosted on a Raspberry Pi, allows teachers to connect to it via a web application from a classroom computer or smartphone. The teacher can configure and modify system settings through the graphical user interface (GUI) provided by the web application. A straightforward algorithm assesses the distance between occupied seats, ensuring compliance with imposed restrictions. Notably, this system prioritizes privacy, distinguishing it from camera-based alternatives.

Meanwhile, the authors of [4] suggest undertaking the text classification task using the two previously mentioned models for two languages (English and Brazilian Portuguese) across distinct datasets. According to their findings, DistilBERT exhibits a training time approximately 45% faster for both English and Brazilian Portuguese compared to its larger counterpart. Furthermore, it is around 40% smaller yet maintains approximately 96% of language comprehension skills, particularly for balanced datasets.

In [5], a multi-delay identification method is proposed based on improved time-correlation analysis. Initially, the data undergo gray relational analysis for preprocessing, leading to the construction of a time delay sequence and a data matrix for time correlation. The multi-delay identification problem is subsequently reformulated as an integer optimization problem. The optimization is performed using an enhanced discrete state transition algorithm to acquire multi-delay. Lastly, to assess its performance, the proposed method is compared with the unimproved time delay identification method and the model without an identification method, utilizing a Neodymium (Nd) component content model constructed by a wavelet neural network. The proposed algorithm enhances optimization accuracy, convergence speed, and stability. The effectiveness of the proposed method is further validated by the significant improvement in the performance of the component content model after time delay identification, particularly in the context of the rare earth extraction process.

The analysis presented in [6] focuses on the attention heat map of benchmarks, revealing that prior models placed greater emphasis on individual phrases rather than capturing the holistic semantic information of the entire sentence. Additionally, a strategy was introduced to disperse attention away from opposing sentiment words, preventing one-sided judgments. A two-stream network was devised, incorporating the gradient reversal layer and feature projection layer within the auxiliary network. The gradient reversal layer was employed to invert the gradient of features during training, optimizing parameters based on the reversed gradient in the backpropagation stage. An auxiliary network was utilized to extract backward features, which were then integrated into the main network along with the standard features obtained by the main network. This approach was implemented across three baseline models—TextCNN, BERT, and RoBERTa—using sentiment analysis and sarcasm detection datasets. The outcomes demonstrated a 0.5% enhancement for sentiment analysis datasets and a 2.1% improvement for sarcasm detection datasets.

The authors of [7] investigate the detrimental effects of packet loss on the video quality encoded using different combinations of compression parameters and resolutions. Their research utilizes a dataset comprising 11,200 full HD and ultra HD video sequences encoded in H.264 and H.265 formats across five bit rates, incorporating a simulated packet loss rate (PLR) ranging from 0 to 1%. Objective assessment relied on peak signal-to-noise ratio (PSNR) and Structural Similarity Index (SSIM) metrics, while subjective evaluation employed the widely recognized absolute category rating (ACR). Their results confirmed the anticipated decline in video quality with an increase in packet loss rate, irrespective of compression parameters. The experiments also revealed that the quality of sequences affected by PLR diminishes with higher bit rates. The paper concludes with recommendations for compression parameters suitable for various network conditions.

The primary goal of [8] was to optimize the conventional 3DCNN model and introduce a novel architecture that integrates 3DCNN with Convolutional Long Short-Term Memory (ConvLSTM) layers. Through experiments conducted on the LoDVP Abnormal Activities dataset, UCF50 dataset, and MOD20 dataset, the results highlight the superior performance of the 3DCNN + ConvLSTM fusion in the realm of human activity recognition. The proposed model is suitable for real-time human activity recognition applications and holds potential for further improvement with the incorporation of additional sensor data. To offer a comprehensive comparison, they evaluate the proposed 3DCNN + ConvLSTM architecture across these datasets. The authors achieved 89.12% precision for the LoDVP Abnormal Activities dataset, and for the modified UCF50 dataset (UCF50mini) and MOD20 dataset, they achieved 83.89% and 87.76% precision, respectively. In summary, their study underscores the efficacy of combining 3DCNN and ConvLSTM layers to enhance accuracy in human activity recognition tasks, positioning the proposed model as a promising candidate for real-time applications.

The authors of [9] outline the development of an Internet of Things (IoT)-based connected university system. Though various smart solutions have emerged at the university, their adoption has been limited among users. The IoT-based connected university system addresses this by facilitating the integration of multiple subsystems, allowing end-users to access diverse solutions through a unified interface. Employing a microservices architecture, the system prioritizes robustness, scalability, and universality. Currently, four subsystems are implemented: indoor navigation, parking assistants, smart classrooms/offices, and news aggregation from university life. The paper comprehensively details the principles governing each subsystem and presents the system’s implementation as both a web interface and a mobile application. A detailed account of the indoor navigation subsystem using Bluetooth beacons is also provided. The paper includes a thorough presentation of the Bluetooth-based indoor navigation concept, considering diverse node placements. Real-world tests were conducted to assess the feasibility of the navigation module, employing deterministic fingerprinting algorithms for precise estimation of users’ device positions.

The research presented in [10] evaluates the usability of several Apple MacBook Pro laptops for basic machine learning research applications, encompassing text-based, vision-based, and tabular data. Four distinct benchmarks were executed, employing four MacBook Pro models—M1, M1 Pro, M2, and M2 Pro. A Swift-script was employed to train and assess four machine learning models utilizing the Create ML framework, in three iterations. The script also recorded performance metrics, particularly time-related outcomes. The findings are presented in tabular form, facilitating a comparative analysis of each device’s performance and the influence of their respective hardware architectures.

The research presented in [11] introduces an inventive data augmentation strategy aimed at identifying distinct student behaviors by leveraging focused behavioral attributes. The primary goal is to alleviate the pedagogical workload. The first step is to curate a concise dataset tailored for discerning student learning behaviors, followed by the application of data augmentation techniques to significantly expand its size. Moreover, the architectural prowess of the Extended-efficient Layer Aggregation Networks (E-ELAN) is harnessed to effectively extract a diverse array of learning behavior features. Notably, integrating the Channel-wise Attention Module (CBAM) focal mechanism into the feature detection network enhances the network’s ability to detect key cues relevant to student learning behaviors, thereby improving feature identification precision. The methodology concludes with the classification of the extracted features through a dual-pronged conduit: the Feature Pyramid Network (FPN) and the Path Aggregation Network (PAN). Empirical evidence vividly demonstrates the potency of the proposed methodology, yielding a mean average precision (mAP) of 96.7%. This accomplishment surpasses comparable methodologies by a substantial margin of at least 11.9%, conclusively highlighting the method’s superior recognition capabilities. This research has significant implications for teaching evaluation systems, reducing the burden on educators while enhancing the objectivity and accuracy of teaching evaluations.

The authors of [12] explore a self-supervised binary classification algorithm designed for defect image classification within ductile cast iron pipe (DCIP) images. Utilizing the CutPaste-Mix data augmentation strategy, they amalgamate defect-free data with enhanced data, feeding them into a deep convolutional neural network. Gaussian Density Estimation is then employed to compute anomaly scores, facilitating the classification of abnormal regions. The proposed approach has been implemented in several real-world scenarios, encompassing equipment installation, data collection, and experimentation. The results showcase the robust performance of the method, which is evident in both the DCIP image dataset and practical field applications, achieving an impressive 99.5 AUC (Area Under Curve). It is a cost-effective method for providing data support for subsequent DCIP surface inspection model training.

In [13], three images—Sentinel-2, GF-1, and Landsat 8—were chosen, and three sample selection methods, namely grouping selection, entropy-based selection, and direct selection, were applied. Subsequently, the selected training samples were utilized to train three supervised classification models—random forest (RF), support-vector machine (SVM), and k-nearest neighbor (KNN). The classification results of the three images were then evaluated. The experimental outcomes indicated similar performances among the three classification models. Notably, the grouping selection method achieved higher classification accuracy using fewer samples compared to the entropy-based method. Furthermore, compared to the direct selection method with an equal number of samples, the grouping selection method exhibited superior performance. Hence, the grouping selection method demonstrated the most favorable outcomes. Additionally, when employing the grouping selection method, the image classification accuracy demonstrated an increase with the augmentation of the number of samples within a specified sample size range.
